# Chemical, Antioxidant, and Antimicrobial Properties of the Peel and Male Flower By-Products of Four Varieties of *Punica granatum* L. Cultivated in the Marche Region for Their Use in Cosmetic Products

**DOI:** 10.3390/antiox11040768

**Published:** 2022-04-12

**Authors:** Maria Rosa Gigliobianco, Manuela Cortese, Samanta Nannini, Lucrezia Di Nicolantonio, Dolores Vargas Peregrina, Giulio Lupidi, Luca Agostino Vitali, Elena Bocchietto, Piera Di Martino, Roberta Censi

**Affiliations:** 1 Percuros B.V., Zernikedreef 8, 2333 CL Leiden, The Netherlands; m.gigliobianco@percuros.nl; 2 CHiP Research Center, School of Pharmacy, University of Camerino, Via Madonna delle Carceri, 62032 Camerino, Italy; manuela.cortese@unicam.it (M.C.); lucrezia.dinicolantonio@unicam.it (L.D.N.); giulio.lupidi@unicam.it (G.L.); luca.vitali@unicam.it (L.A.V.); roberta.censi@unicam.it (R.C.); 3 ABICH Srl, Biological and Chemical Toxicology Research Laboratory, 28924 Verbania, Italy; samanta.nannini@hotmail.it (S.N.); elena.bocchietto@abich.it (E.B.); 4 Recusol Srl, 62032 Camerino, Italy; dolores.vargas@unicam.it; 5 Department of Pharmacy, Università “G. D’Annunzio” Chieti e Pescara, Via dei Vestini, 1, 66100 Chieti, Italy

**Keywords:** agri-food waste, pomegranate wastes, pomegranate’s flower by-products, green extraction, antioxidant capacity, UPLC-ESI-MS/MS analysis, antimicrobial activity

## Abstract

We are now seeing an increase in the production of agri-food waste, which is an essential resource for the recovery of bioactive compounds that may be employed as innovative natural ingredients in cosmetics. To date, the approach to cosmetics preservation has seen a significant shift in the search for biological components that give healthier alternatives for customers and help businesses operate in an environmentally friendly manner. To achieve this goal, we studied pomegranate extracts using the peel and, for the first time, extracts from the male flowers of a wide pomegranate variety cultivated in the Marche region, specifically, the Wonderful, Mollar de Elche, Parfianka, and less-studied G1 varieties. We studied the phenol compounds profile, antioxidant capacity, antimicrobial activity, and cell viability of the obtained pomegranate extracts. The identification and quantification of phenol compounds belonging to different classes, such as hydrolysable tannins, hydroxybenzoic acid, hydroxycinnamic acid, dihydroflavonol, gallocatechin, and anthocyanins, were performed using UPLC-ESI-MS/MS. Punicalagin isomers and punicalin resulted in the most abundant polyphenols found in the peel and male flower extracts. Mollar de Elche 2020 peel extract revealed a high concentration of punicalagin A and B (7206.4 mg/kg and 5812.9), while the content of gallic acid revealed high results in the G1 and Parfianka varieties. All extracts were spectrophotometrically analysed to determine their total phenol content (TPC) using the Folin–Ciocalteu method and their antioxidant capacity (AC). In terms of the total phenol obtained by the Folin–Ciocalteu colorimetric method, Mollar de Elche 2020 extracts reported the highest TPC content of 12.341 µmol GAE/g. Results revealed that the Mollar de Elche and Wonderful 2020 peel extracts demonstrated the highest TPC and AC. Furthermore, AC results indicated that the peel extracts displayed higher AC than the male flower extract due to the high punicalagin content detected by UPLC analysis. The antimicrobial activity testing revealed that the Wonderful and G1 2020 peel extracts resulted active against *Escherichia coli*, while all extracts exhibited promising anticandidal activity. Additionally, the cytocompatibility was evaluated in keratinocytes HaCaT cells by testing concentrations of pomegranate extracts ranging from 0.15 to 5.00 mg/mL. Extracts were non-toxic for the cells in the tested concentration range. The acquired results may help exploit pomegranate agri-food waste products provided by the Marche region’s short supply chain for their use as an antimicrobial and antioxidant booster in the formulation of cosmetic products.

## 1. Introduction

In recent years, the cosmetics sector’s new focus has been on two crucial challenges: ecology and sustainability. Given this sustainable approach, companies are currently using natural ingredients to enhance the quality of the cosmetics, keep them free of microorganisms contamination, enhance their shelf-life, obtain safer products, and avoid the use of synthetic preservatives. 

Furthermore, the typical approach to cosmetic preservation is to employ the most significant number of conventional preservatives allowed (refer to Annex V of the European Commission’s Regulation (EC) No 1223/2009 for a list of authorized preservatives [1]). Conventional preservatives have shown many secondary effects and risks on human health. Recent studies have also reported side effects from preservatives on the skin microbiota [2,3]. The use of cosmetic products has recently been shown to change the equilibrium of the skin microbiota, which can cause it to lose its healthy status. This effect can be linked to various causes, including the preservatives’ residual impact on the skin. 

Against this background, the approach to cosmetics preservation can be managed with several strategies to prevent microbial contamination. Various methods can be used to achieve these goals. One solution is based on the concept of “Hurdle Technology”, which encompasses all intelligent ways of preventing and reducing microbe proliferation throughout the formulation production process. As a result of this approach, several steps must be taken to avoid microbial contamination, ranging from strict adherence to GMP, raw material microbial control, and water treatment using a different strategy to reduce water activity, as water is one of the most abundant ingredients in many cosmetic formulations. In adherence to GMP, qualified employees can manage cross-contamination, disinfection of equipment, and proper packaging. (e.g., airless dispensers, pumps) are essential [4]. Any of these measures, when combined, can help lower any microbial contamination.

On the other hand, using novel raw materials obtained from agri-food waste is one way to avoid using conventional preservatives and it depends more and more on innovative ingredients. This strategy provides healthier options for customers and assists businesses in working in an ecologically sustainable manner. This method is also feasible since it is widely recognised that some natural ingredients may play many roles in cosmetic formulation, such as absorbed water making a formulation alkaline or acid. They can also be applied as a natural antibacterial. Herbal extracts and essential oils have shown evidence of microbial activity despite not being preservatives but also exhibiting antimicrobial activity with double safety rules: safety of the formula, where we do not want microorganisms, and protection of the skin, where we do not want side effects [5]. Currently, a feasible strategy for reintroducing agri-food waste into natural cosmetics manufacturing is the circular economy [6,7,8]. Natural compounds obtained from agri-food waste products can be used successfully as a natural preservative in cosmetic compositions [9,10]. One of the most interesting by-products that may be utilised for this purpose is pomegranate by-products, which are becoming recognised as having numerous bioactive compounds with considerable antioxidant activity and antibacterial properties [11].

Pomegranate, or *Punica Granatum* L., one of the most ancient fruits, is a fruit that belongs to the family Punica and has many benefits to human health. It is grown worldwide with approximately 1500 ha of cultivars in Italy, mostly in Sicily, Puglia, Calabria, Campania, and Lazio [12]. Still, many of these cultivations are also present in the Marche region. In the last decade, the interest in the cultivation of *Punica Granatum* L. also highlights the varieties that have encouraged the revival of pomegranates in Italy as an income crop. Therefore, many entrepreneurs, driven by the need to diversify and expand their business, have identified a viable alternative to traditional orchards. Consequently, this situation has led to a significant increase in waste. Usually, pomegranates generate 669 kg of waste materials for every 1 ton of fresh fruit, with 78% composed of peel and 22% seed [13,14]. Different studies report pomegranate peel as an interesting by-product [12,15,16], but we also identified another part of the pomegranate tree that provides an abundant waste material. During the flowering period, from May to July, the pomegranate tree develops two principal types of flowers: male flowers and hermaphrodite flowers. The first one has a poorly designed or no pistil and atrophied ovaries with few ovules. It is infertile and drops down without fruit set, representing considerable waste material. In contrast, the hermaphrodite flowers are fertile, with an ovary producing fruit. The primary by-products of the pomegranate flower, the male ones, have not been investigated singularly as potential sources of bioactive polyphenols. To the best of our knowledge, no data are available.

Pomegranate peel and flowers represent an enormous waste of raw materials. As reported by the literature, pomegranates contain a high number of phenol ingredients in their pulp, seed, and juice [17,18,19,20]. Polyphenols represent the predominant phytochemicals of the pomegranate peel and are mainly composed of hydrolysable tannins (pedunculagin, punicalin, punicalagin, and ellagic acids). Among them, the predominant hydrolysable tannin of pomegranates is known as the punicalagin [21]. In addition, there are also other flavonoids such as quercetin, kaempferol, luteolin glycosides, catechins, anthocyanins, and complex flavonoids [20,22]. These bioactive compounds are responsible for many pharmacological properties, notably their significant antioxidant activity and antibacterial characteristics [18,23]. Total phenolic content, antioxidant capacity, and antibacterial activity against various microbes have been highly correlated [24]. Pomegranate peel extracts and other fruit by-products, such as juice or seeds, have exhibited remarkable antioxidant and antibacterial properties, with a high scavenging capacity, reducing the microbiological proliferation of several bacteria, among other therapeutic benefits to the human body [25].

Due to their high content of ellagitannins, such as punicalagin, *Punica Granatum* L. peel extracts show a remarkable antimicrobial capacity influenced by the different types of cultivars, which has been demonstrated against pathogenic such as *Staphylococcus aureus* [26]. The antimicrobial activity was associated with a high concentration of punicalagin and an ellagic acid concentration in the pomegranate peel extract, demonstrating that the ellagic acid content substantially impacts the antimicrobial activity [27,28,29].

Based on the potential benefits of pomegranate extract and the need for research exploring natural ingredients that may be used as alternative preservatives in cosmetic formulations, our study aims to evaluate the antioxidant capacity and anti-microbial activities of extracts of the peel and male flower by-products of four pomegranate varieties cultivated in the Marche region. The US green extraction processes were applied to efficiently recover the polyphenols from the pomegranate peel and male flower extract. The main polyphenol compounds present in the peel and male flower extracts were identified and quantified by UPLC-ESI-MS/MS. The majority of the data on pomegranate extracts in the literature came from one of the most common varieties. As a result, our research presents a chemical and antibacterial analysis of pomegranate varieties that have received less attention in the past, such as G1 and Parfianka. To our knowledge, the characterisation of polyphenols and antibacterial activity for the combination of pomegranate and other fruits, such as the G1 cultivar, has never been studied. As a result, this study will provide a scientific foundation for supporting the valorisation of pomegranate peels and male flower by-products received through a short supply chain as a substantial source of natural preservatives used in the production of cosmetics with a reduced rate of conventional ones. To achieve this goal, pomegranate extracts might be used as a preservative booster with antimicrobial properties, decreasing the need for synthetic preservatives in cosmetics and as a booster fulfilling other specific purposes, such as moisturiser or antioxidants.

## 2. Materials and Methods

### 2.1. Chemicals

The chemicals 1,1-Diphenyl-2-picrylhydrazyl (DPPH), 2,4,6-Tris(2-pyridyl)-s-triazine (TPTZ), (±)6-Hydroxy-2,5,7,8-tetramethylchromane-2-carboxylic acid (TROLOX), 2,2′-Azino-bis(3-ethylbenzothiazoline-6-sulfonic acid), diammonium salt (98%TLC), ABTS, gallic acid, sodium carbonate monohydrate ACS reagents, and ethanol (final ethanol grade) were purchased from Sigma-Aldrich (Steinheim, Germany); Manganese (IV) oxidise activated (˃90%), and Folin–Ciocalteu’s phenol reagents were purchased from Fluka (Buchs, Switzerland). Analytical grade solvents and reagents were used for all of the work. Ellagic acid, gallic acid, punicalagin A and B, punicalin, cyanidin 3-glucoside, cyanidin 3,5-diglucoside, pelargonidin 3,5-diglucoside, and pelargonidin 3-glucoside were obtained from Sigma-Aldrich (Stenheim, Germany). Formic acid and acetonitrile for LC/MS were purchased from Carlo Erba Reagents (Cornaredo, MI, Italy). Sigma Aldrich provided all the other chemicals (Steinheim, Germany). The ultrapure water was produced from the Millipore system (Millipore Sigma, Darmstadt, Germany), and filtered with a 0.20 μm Sartolon polyamide filter (Sartorius Stedim Biotech, Göttingen, Germany).

### 2.2. Pomegranate Samples

In this study, Wonderful, Mollar de Elche, Parfianka, and G1 pomegranate cultivars, grown in the Marche region, were harvested in October 2019 and 2020 at commercial maturity at the agricultural enterprise “Onori Maria Rosaria”, Sant’Elpidio a Mare (Fermo, Marche). Immediately after the fruits were collected, the pomegranate peels were manually removed to accurately separate the exocarp (rind) used for the extraction process from the mesocarp (white spongy tissue). The exocarp was dried at 37 °C in a ventilated oven (VEC2103/8, Everest, Rimini, Italy). The male flowers, and more specifically sepals and stamen, of Wonderful and Mollar de Elche were picked during the flowering period, in June 2020, from the same agricultural enterprise from which we obtained all of the materials for our investigation. The waste male flowers were promptly dried in a ventilated oven at 37 °C until they reached a consistent weight and were then utilised for extraction. For each sample, three extractions were performed.

### 2.3. Extraction of the Phenol Compounds from Pomegranate Peel and Male Flowers 

Extractions from the pomegranate peel and male flowers were carried out in an ultrasonic bath (FALC-LABSONIC LBS2, Treviglio, BG, Italy), with a maximum capacity of 10 L. Extractions were performed using a water and ethanol at 70:30 (*v*/*v*) mixture as extraction solvent. The plants (1 g) were dispersed in 100 mL of solvent and then immersed in an ultrasonic bath (50 °C, 59.2 W/cm^2^ for 15 min). The extracts were lyophilised at −53 °C for 24 h at 0.03 millibar (FreeZone, 1 L, LABCONCO, Kansas City, MO, USA). Each sample was kept at −20 °C in 50 mL screw-top cap polyethylene vials (BD Falcon TMBD Biosciences, Bedford, MA, USA). The lyophilised solids were reconstituted with appropriate solvents for subsequent investigations.

### 2.4. Ultra-Performance Liquid Chromatography Mass Spectrometry Analysis

UPLC (Agilent 1290 Infinity Technologies UPLC, Santa Clara, CA, USA) with a diode array detector and a triple quadrupole mass spectrometer (MS-QQQ), combined with an electrospray ionization source (ESI), was used to identify and quantify polyphenols in pomegranate extracts. The UPLC had a binary pump and an auto-sampler. We used a Luna (C18 1504.6 mm) column (Phenomenex, Castel Maggiore, BO, Italy) at 25 °C for chromatographic separation. The gradient elution used water/0.1% formic acid (solvent A) and acetonitrile/0.1% formic acid (solvent B) at a 1 mL/min flow rate. The optimised gradient was 0–3 min, 7% B, 3–30 min, 27% B, 30–60 min, and 100% B, and 5 µL was injected. The mass spectrometer detection in both negative and positive ionisation modes was utilised to identify the different signals. However, in the negative mode, a significant group of compounds matching deprotonate molecular ions of diverse chemicals were identified.

In contrast, in the positive mode, the characteristics of anthocyanins of pomegranate were detected. The peak characterisation was performed based on their retention time and accurate molecular mass in the MS and MS^2^ experiment. The ESI source worked at 350 °C, 12 L/min, and a nebulizer pressure of 55 psi. Samples were diluted 1:2 with mobile phase, sonicated for 5 min, then centrifuged at 12,000 rpm for 10 min (Scilogex D3024R High-Speed Refrigerated Micro-Centrifuge, Rocky Hill, CT, USA). Next, a 0.20 µm filter (Captiva Econofilter, PTFE) was used to filter the samples. A full scan mass spectrometer analysis was performed in *m/z* 150–1500 (negative) and 50–1500 (positive). Following that, several production experiments were conducted on the corresponding precursor ion. The quantification of polyphenols was carried out for individual phenol compounds using a calibration curve of the respective reference external standards. To this end, the mixed standard was prepared at 1000 mg/L. The linearity, sensitivity, accuracy, and precision of the developed method were verified according to the Food and Drug Administration Guidelines (FDA), as already mentioned in our previous studies [30]. The linearity was explored in the concentration range of 1–50 mg/L for ellagic acid, gallic acid, punicalagin A and B, and punicalin. External anthocyanins standards as cyanidin 3-glucoside, cyanidin 3,5-glucoside, pelargonidin 3,5-glucoside, and pelargonidin 3-glucoside were used with a concentration range of 1–20 mg/L, obtaining the corresponding calibration curve for further calculations (Table 1). The performances of this method were also tested for accuracy, precision, and sensitivity (Table 1). The sensitivity was evaluated in terms of the limit of detection (LOD) and limit of quantification (LOQ) by triple injections of a standard mixture at concentrations responsible for a signal-to-noise ratio equal to 3 and 10 for LOD and LOQ, respectively. To evaluate the method’s accuracy, we chose a concentration of 10 mg/L for all standards. They were injected in triplicate among the unknown samples in the daily sequence. The accuracy was expressed as standard deviation percentage and reported in the Supplementary Materials. The overall method precision was evaluated by analysing nine extracts of the same sample. Each section was then injected individually as an unknown sample. The accuracy was calculated for each compound and expressed as a relative standard deviation percentage.

The matrix effect (ME) for the matrices used in this study was also evaluated. The Matrix Effect percentage (ME%) was calculated using this Equation (1):

(1) $$\mathrm{ME}\%=\frac{B}{A}\times 100\;$$

where *B* is the difference between the areas of the fortified sample and non-fortified one, and *A* is the area of the standard solution in the pure solvent. Thus, it was possible to evaluate if the matrix effect induced an ion suppression (values < 100%) or an ion enhancement (values > 100%). A ME value equal to 100% means that no matrix effect occurs; deals over 100% reveal a signal enhancement and matters less than 100% reveal a signal suppression [31].

### 2.5. Determination of the Total Phenol Content

Total Phenol Content (TPC) was determined by using the Folin–Ciocalteu spectrophotometric method according to Gigliobianco et al. [30,32,33]. The results were expressed as milligrams of gallic acid equivalents (GAE) per grams of by-product (mg GAE/g).

### 2.6. Antioxidant Capacity (AC) Evaluation

Three methods were adopted in our approach to measure antioxidant activity: DPPH• radical scavenging activity, 2,2′-and-bis (3-ethylbenzothiazoline-6-sulphonic acid) (ABTS•+) radical cation scavenging capacity, and Ferric Reducing Antioxidant capacity (FRAP) [22,23]. Trolox, which is 6-hydroxy-2,5,7,8-tetramethylchroman-2-carboxylic acid, was used as a standard. Results were shown in terms of IC50, which is the concentration of the test sample that would reduce the concentration of DPPH, ABTS by 50%, as well as the amount of Trolox equivalent (TE)g-1 in the sample.

The DPPH free radical scavenging activity was assessed using previously published methods with slight modifications [34,35]. In a 96-well microliter plate, 100 µL of the sample (10 mg mL^−1^) and standard were added to 150 µL of DPPH in 100 ethanol. At 517 nm, the absorbance of each well was measured after 20 min of incubation at 37 °C.

The ABTS analysis was carried out as described in [36] using a 96-well microliter plate [35]. We made the ABTS•+ solution by oxidizing it with MnO_2_ in water for 30 min. The ABTS•+ solution (150 µL) was added to a 50 µL aliquot of the different concentrations of the sample (concentration 10 mg mL^−1^) and standard (Trolox). After 10 min of incubation at room temperature, the absorbance of each well was measured at 734 nm.

The FRAP values of the extracts were calculated using a previously described technique [37], with minimal modifications [38]. The FRAP reagent was produced by dissolving the following three solutions: 50 mL 0.3 M acetate buffer pH 3.6 (1.23 g sodium acetate in 50 mL water acidified with acetic acid); 5 mL stock solution of 5 mM TPTZ (2,4,6-tripyridyl-s-triazine) (15.6 mg) in 40 mM HCl; 5 mL stock solution of 5 mM FeCl3 6H2O (16.2 mg) in 40 mM HCl. The FRAP solution was warmed to 37 °C. Aliquots of 50 µL sample solution (10 mg mL^−1^) were applied in triplicate to the 96-well plate (BD FalconTM). Each well was initiated with 175 µL of FRAP reagent. The plate was immediately shaken for 30 s in a FLUOstar Omega plate reader, and the reaction was allowed to run for 10 min before being read on a plate reader (593 nm). Meanwhile, a reference solution of Trolox was run to create the calibration curve by linear regression with the linearity of R^2^ = 0.9996. The data were presented in µM Trolox equivalent (TE) g-1 sample.

### 2.7. In Vitro Antimicrobial Activity Assay

Extracts were tested against the Gram-negative bacterial species Pseudomonas aeruginosa (ATCC 9027) and Escherichia coli (ATCC 8739), the Gram-positive Staphylococcus aureus (ATCC 6538), and the yeast Candida albicans (ATCC 10231). These microorganisms were from the culture collections maintained by the In Vitro Testing Laboratory of Abich s.r.l. (Verbania, VB, Italy).

The raw materials of the pomegranate peel and male flower extracts were initially evaluated for their TVC (total viable counts) according to ISO 21149 for bacteria and ISO 16212 for yeast and moulds. The bacterial TVC were < 10 UFC/g for all analysed pomegranate cultivars. Only male flowers had a bacterial TVC > 10 UFC/g, probably because the flowers were collected directly from the field after they had fallen from the tree. No contamination was detected in the yeast and moulds except for the Mollar de Elche peel (2019 and 2020). Furthermore, we also found no microbiological contamination in pomegranate extracts. The antimicrobial activity of extracts was evaluated by following the ISO 20645 based on an agar-disk diffusion method with some modifications for *C. albicans* and the filter paper size used. Petri dishes were prepared with a bottom layer of about 10 mL of a non-inoculated agar medium, which was Tryptone Soy Agar (TSA) (VWR, Milano, Italia) for bacteria and Sabouraud Dextrose Agar (SDA) for Candida. Then a top agar inoculated with 10^6^ CFU/mL of cells was poured onto the bottom layer. After solidification, a sterile filter paper disc (about 6 mm in diameter) containing the test compound was placed on the surface of the inoculated top agar. The disks were prepared by soaking up 100 µL of extract and then placing them still wet on top of the agar layer. Negative control disks were soaked in the solvent used for the extraction. Plates were incubated at 37 °C for 24 h. To assess the antimicrobial activity of the extracts, bacterial and yeast growth inhibition under and around the disk was evaluated, and the diameter of the inhibition zone was measured and given in millimetres. The growth inhibition was calculated on a scale of 0% to 100%, with 0% indicating no inhibition and 100% indicating the complete absence of growth below the disk. Each experiment was repeated three times.

#### Effect of Pomegranate Extracts on Bacterial Growth

The susceptibility of the four microorganisms to different concentrations of pomegranate extracts was determined by the broth microdilution method, and the inhibition of growth was assessed by a turbidimetric assay. A serial dilution of the extracts (highest concentration: 50 μg/μL) was prepared in a growth medium and inoculated with bacteria at 37 °C for 24 h. A calibration curve correlated the turbidimetric values with bacterial cell counts. Fertility and sterility tests were run in parallel with ranging concentrations of Tryptone soy broth (TSB) (VWR, Milano, Italia) and extracts, respectively. After the incubation at 37 °C for 24 h, the optical density at *A*_600nm_ was determined, and the percentage of inhibition was obtained by the following Equation (2):

(2) $$Inibition\left(\%\right)=100-\frac{O.D./mL{\;}_{extract}}{O.D./mL{\;}_{no\;extract}}\times 100\;$$

### 2.8. Cell Viability Evaluation of Pomegranate Extracts in Keratinocyte Cells

The human keratinocyte cell line HaCaT was cultured at 37 °C in a humidified incubator with 5% CO_2_ in Dulbecco’s Medium (DMEM) supplemented with 10% foetal bovine serum, two mM L-glutamine, 50 U/mL penicillin, and 50 g/mL streptomycin. To assess the intracellular antioxidant activity of HaCaT cells, they were seeded at a density of 3104 cells per well in 96-well plates. The studies were conducted 24 h after incubation at 37 °C in 5% CO_2_.

Cell viability was determined by decreasing MTT as described in our previous work [30]. At 37 °C in 5% CO_2_, HaCaT cells were treated for 24 h with various concentrations of extracts (0.160–5 mg/mL). The treatment medium was changed to MTT in HBSS (0.5 mg/mL) for two hours at 37 °C in 5% CO_2_. Formazan crystals were dissolved in isopropanol after being washed with HBSS. The formazan concentration was determined (570 nm, reference filter 690 nm) using the VICTOR^TM^X3 multilabel plate reader (PerkinElmer, Waltham, MA, USA). The viability of the cells was expressed as a percentage of total vitality.

### 2.9. Statistical Analysis

As appropriate, we used one-way ANOVA together with Dunnett or Bonferroni post hoc tests to conduct our statistical study. On a Windows platform, analyses and Pearson correlation analysis were performed using GraphPad PRISM software (version 5.0; GraphPad Software, La Jolla, CA, USA).

## 3. Results and Discussion

### 3.1. Qualitative Polyphenols Identification

We obtained the mass data in the full-scan analysis, and product ion mass data were acquired by the information-dependent acquisition method. Thirty-one phenol compounds, including hydrolysable tannins such as ellagitannins and gallotannins, and phenol acids from the peel extract of pomegranate, were identified based on previous studies [39,40,41,42,43]. Compounds were observed by their [M − H]^−^ ions in negative ESI mode and their [M]^+^ ions in positive mode, which was helpful to detect their precise mass measurement. To organize the description of peak assignment and further characterization of individual compounds, all the phenol compounds were divided according to their compound classes in hydrolysable tannins, hydroxybenzoic acids, hydroxycinnamic acids, and dihydroflavonol for the portion of the negative ion. While for positive ions, four anthocyanins were identified. To obtain the most valuable chemical information and achieve better attribution of every peak of specific substances, G1 peel extract was selected out of all the extract samples for the investigation.

A comprehensive description of the method adopted for identifying all the substances detected by UPLC-MS/MS can be found in the Supplementary Materials (Tables S1 and S2). Accurate mass measurements, retention time, and main MS/MS product ions for all phenol compounds were reported in the Supplementary Materials.

### 3.2. Quantitative Analysis Using UPLC-ESI-MS/MS

**Figure 1:**
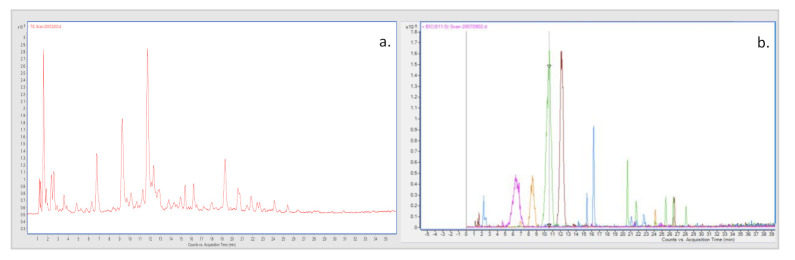
HPLC chromatographic profile of the phenol compounds (**a**), and quantified anthocyanins (**b**) (A4 in brown, A2 in green, A3 in orange, A1 in fuchsia), present in pomegranate peel extracts (variety G1). For peaks identification see Tables S1 and S2.

The methodology established in this research may be used for the quantitative measurement of secondary metabolites contained in the peel and male flower extracts of four different pomegranate varieties. Quantitative data were expressed as mg/kg dry weight matter for all extract samples. Selected polyphenol compounds were quantified and compared among pomegranate peel and male flower extracts. The contents of the nine chosen compounds (gallic acid, punicalin, punicalagin A and B, ellagic acid, cyanidin 3,5-diglucoside, cyanidin 3-glucoside, pelargonidin 3,5 diglucoside, pelargonidin 3-glucoside) in the peel and male flower extracts of pomegranate from four different varieties of pomegranate are shown in Table 2 and Figure 1.

Among phenol compounds, ellagitannins represent the predominant class of phenol compounds present in pomegranate peel and flowers [15,40]. The ellagitannins, punicalagin A and B, punicalin, and ellagic acid were quantified in our work. High levels of punicalagin A and B (7206.4 mg/kg and 5812.9 mg/kg, respectively) were detected in the Mollar de Elche 2020 peel extract. The content of punicalagin A was also high in the Parfianka (3767.3 mg/kg) and G1 (3622.3 mg/kg) 2020 peel extracts. The Mollar de Elche 2019 peel extract (2176.7 mg/kg) and the Wonderful 2020 peel extract (2754.8 mg/kg) revealed a content of punicalagin A higher than the other pomegranate varieties taken into account in this study. The Parfianka peel extract reported a high content in punicalagin B (5367.8 mg/kg), followed by the peel extracts of Mollar de Elche 2019 and Wonderful 2020. Our results are consistent with previous research that found extremely high levels of punicalagin (10,543.4 mg/g) in pomegranate peels. [13,15,44]. The punicalagin in pomegranate peel extract represented the most dominant component in the extract compared with ellagic acid, gallic acid, catechin, and epicatechin [45].

A high concentration of punicalin was detected in the Mollar de Elche 2020 peel extract, followed by the Wonderful and G1 2020 peel extracts. The Wonderful peel extract demonstrated a higher ellagic acid content than the other pomegranate varieties. The ellagic acid in the Wonderful 2020 peel extract was 428.9 mg/kg, while in the Mollar de Elche and G1 2020 peel extracts it was 289.7 and 337.3 mg/kg, respectively. The peel extract of Mollar de Elche 2019 resulted in an ellagic acid concentration of 231.2 mg/kg, while in the Wonderful and G1 peel extracts, the concentration of ellagic acid was lower.

Among the male flower extract, Wonderful 2020 showed a high level of punicalin followed by punicalagin B and A. In Mollar de Elche, the content of punicalin was higher compared to the concentration of punicalagin A and B. In the male flower extract, the ellagic acid content was low for Wonderful (42.4 mg/kg) and Mollar de Elche (87.1 mg/kg). Our work investigated the content of gallic acid, which belongs to the hydroxybenzoic acid class. Among the peel extracts, the samples with a high gallic acid content were the G1 and Parfianka 2020 peel extracts, followed by the Mollar de Elche 2020 and 2019 peel extracts. The concentration of gallic acid in male flower extract of Wonderful showed a high concentration of 925.2 mg/kg compared to the concentration obtained for the Mollar de Elche male flower extract (789.5 mg/kg). The concentrations of phenols detected in our study matched those observed in previous studies. The range of punicalagin in the peel extract was higher than the range of ellagitannins and hydroxybenzoic acids [46,47]. We also investigated the content of anthocyanins which revealed differences in their contents in different cultivars. In detail, the amount of cyanidin 3,5-diglucoside was 25.2 mg/kg in the Wonderful 2019 peel extract, while in the 2020 variety, the concentration was much lower (4.7 mg/kg). This compound was also detected in the Wonderful male flower extract, where the concentration was 6.1 mg/kg, while in Mollar de Elche peel and male flower extract, the concentration of cyanidin 3,5-diglucoside and cyanidin 3-glucoside was under the limit of quantification. Pelargonidin 3,5-diglucoside was detected in high concentration in G1 2019 peel extract (20.6 mg/kg), although, in the G1 2020 peel extract the concentration was under the detection limit. In the Wonderful and Mollar de Elche male flower extract, the pelargonidin 3,5-diglucoside was 2.4 mg/kg and 8.1 mg/kg, respectively. The mono glycoside pelargonidin was 13.1 mg/kg in the Wonderful 2019 peel extract, followed by Parfianka 2020 peel extract and Mollar de Elche 2020 and 2019 peel extracts. In the male flower extract, the content of pelargonidin 3-glucoside was 1.1 mg/kg for the Wonderful variety, while it was under the limit of detection in Mollar de Elche.

Our results confirmed that the phenol composition of pomegranate is strongly influenced by the fruit part (such as peel, mesocarp, and arils), cultivar, environmental conditions, solvent, and methods used for the extraction, as also reported in other studies [21,48,49]. Additionally, our work used a green extraction technique based on US technology, one of the most advantageous greenways for bioactive component extraction. This technology employs cavitation to collapse membrane cells, enabling the extraction of many molecules. The US technology provides various advantages, including low prices and the capacity to reduce extraction time, solvent use, and energy consumption [16,21,50,51,52]. Many studies highlight how US technology is used mainly on natural products, including pomegranate phenol extraction [12,16,21,48,51,53].

### 3.3. The Total Phenol Content (TPC) and Antioxidant Capacity (AC) of Pomegranate Extracts

Table 3 reports the TPC and AC results obtained for the four pomegranate cultivars and their peel and male flower extracts. The Folin–Ciocalteu colorimetric method determined total phenol content and demonstrated impacts that ranged between 0.50 to 12.34 µmol GAE/g for peel extracts, and 0.46 and 0.778 µmol GAE/g for male flower extracts. Among the analysed samples, the peel extract from Mollar de Elche 2020 reported content of 12.341 µmol GAE/g, which represented the highest TPC, while Wonderful 2019 showed the lowest TPC. For the male flower extract samples, the TPC results were 0.778 and 0.746 µmol GAE/g for Wonderful and Mollar de Eche, respectively. The results show that pomegranate peel extracts have a phenol content 20 times higher than the corresponding extracts obtained from the male flowers of the same cultivars of Wonderful and Mollar de Elche. Our results are in line with the TPC of pomegranate peel extract reported in other works [54,55]. Based on the obtained results, we might highlight the impact of the type of cultivars and seasonal variation genotype of TPC in the biosynthetic pathway, as reported in previous studies [26,56,57,58]. The antioxidant capacity was obtained by two methods based on the evaluation of the free-radical scavenging capacity of the peel and male flower extract (DPPH and ABTS) and one method based on the reducing power of the extract samples (FRAP).

To determine whether the pomegranate extracts produced had bio-active antioxidant properties due to their high content of phenol compound, which is widely known as being responsible for antioxidant activity, the in vitro antioxidant activity of all extracts was evaluated using the DPPH, ABTS, and FRAP assays [59]. Our results show that pomegranate peel and male flower extracts display variability in inhibitory activity against DPPH radicals ranging between 0.242–34.361 µmolTEA/g. Among the tested pomegranate peel extract, the highest radical scavenging activity was detected for the Wonderful 2020 peel extract, followed by the G1 2020 peel extract and the Parfianka 2020 peel extract. Meanwhile, male flower extracts show their highest inhibitory activity against DPPH radicals in the following order: Wonderful ˃ Mollar de Elche.

Our work also evaluated the antioxidant activity by ABTS radical cationic decolorization assay, showing that the highest radical activity was detected for the Wonderful 2020 peel extract (29.301 µmol TEA/g) the lowest one in the Wonderful 2019 peel extract. Among male flowers, it was found that the antioxidant activity was 6.808 and 3.168 µmolTEA/g for the Wonderful and Mollar de Elche male flower extracts, respectively.

The ferric reducing and antioxidant power assay was employed. The FRAP results of pomegranate peel and male flower extract were determined to compare the four varieties of pomegranate in our investigation. The Mollar de Elche 2020 and G1 2020 peel extract reported the highest FRAP value among all peel extracts tested, followed by Wonderful 2020, Parfianka 2020, G1 2019, Mollar de Elche 2019, and Wonderful 2019.

We found a positive and high Pearson correlation with a significant *p* value (* *p <* 0.05; **** *p* < 0.0001) between antioxidant activity measured with FRAP and ABTS and total phenolic contents, suggesting that the antioxidant activity is dependent on the number of phenolic compounds present in the extracts.

Results show that the peel extracts displayed higher AC than the male flower extracts. The AC found in pomegranate peel extracts was connected with the punicalagin concentration determined by our quantitative study, and it was also consistent with other studies [15,55].

### 3.4. Antimicrobial Activity Evaluation 

To explore the possibilities of using pomegranate by-products as a natural preservative in cosmetic formulations alone or as a booster to reintroduce agri-food waste into the cosmetics formulation as a natural bioactive component, we investigated the antimicrobial activities of peel and male flower extracts derived from Wonderful, Mollar de Elche, Parfianka, and G1 cultivars. The evaluation of our extracts’ activity against the Gram-positive and Gram-negative was obtained by the disk-diffusion testing [60] (Table 4). The extracts activities were tested against *Staphylococcus aureus, Escherichia coli, Pseudomonas aeruginosa,* and *Candida albicans* which are microbial species referenced in the challenge test for cosmetic products. The Wonderful 2020 peel extract was the most efficient against *Staphylococcus aureus*, with a 70% growth inhibition in microdilution tests and an inhibition zone diameter of 3 mm by disk diffusion. The highest antimicrobial activity against the *Escherichia coli* was demonstrated by the Wonderful 2020 and G1 2020 peel extracts, while all cultivars were active against *Candida albicans*. In particular, a good activity was detected for the G1 2019 and 2020 peel extracts, the Wonderful 2020 peel extract, and the Mollar de Elche 2020 peel extract. The antimicrobial activity obtained for peel extracts was in line with a previous work by Khan et al. [61] reporting high antimicrobial activity for pomegranate peel extract against Gram-negative bacteria. Besides, Kupnik et al. [62] found that *E. coli* and *P. aeruginosa* were more susceptible to pomegranate peel extract than to other parts of the fruit. In accordance with other studies, peel and male flower extract were effective also against Gram-positives [5,26,62,63,64].

The peel G1 extract was chosen as the most promising among the tested extracts for further antibacterial investigation using a turbidimetric test. Table 5 shows the IC_50_ obtained against *S. aureus* and *P. aeruginosa* in G1 2020 peel extract. It was detected that a 1:2 dilution of the extract was effective in reducing 97% of the bacteria.

Our pomegranate extract demonstrated a variable antimicrobial activity against the four microbial species considered. This might be attributed to the pH values ranging from 3.9 ± 0.2 to 4.6 ± 0.3 that were found in all of the extracts and the high concentration of polyphenols that can inhibit microbial growth. Data herein indicated that peel extract, mainly from the G1 and Wonderful varieties, had an effective antimicrobial activity, due to its inhibitory effect on bacterial growth. At a concentration of 10 mg/disc, the G1 extract induced the formation of a clear inhibition zone ranging from 1 to 4 mm against all microorganisms tested. Another extract with a measurable antimicrobial activity was the Mollar de Elche 2020 peel extract, with an ability to inhibit the growth of the *Aspergillus brasiliensis* (data not shown). Furthermore, this conclusion is supported by the TVC of the yeasts and moulds, which were found lower than 10 UFC/g in raw materials from the Mollar de Elche 2019 and 2020 peel extract.

Our results concur with several studies on the antimicrobial activity of pomegranate extracts [5,26,64].

Accordingly, we can correlate the antimicrobial activity of pomegranate peel extract with the high concentration of polyphenol compounds such as punicalagin and punicalin as evidenced by the UPLC-ESI-MS/MS analysis. The pomegranate peel extract obtained from the G1 cultivars demonstrated interesting inhibitory activity and had among the highest concentrations of punicalagin (3622.3 and 2805.7 mg/kg for two isomers A and B, respectively). Punicalagins use their hydrophilic part to interact with the polar regions of the cell membrane compromising its ability to transport substances inside the cell [5,63]. Additionally, phenols can render substrates unavailable to microorganisms or interfere with protein secretion.

The results against the Gram-negative for the G1 and Wonderful pomegranate extracts pave the way for their further application as booster antimicrobial agents to be used in combination with common synthetic antimicrobials, which might be added in lower amounts thereby increasing skin tolerance and product safety. Furthermore, since these extracts are obtained from the peel and male flowers of pomegranate, they provide a concrete example of natural products made from agri-food waste that have been reintroduced into the cosmetics’ circular economy concept.

### 3.5. Cytocompatibility of Pomegranate Extract in Human Keratinocytes

**Figure 2:**
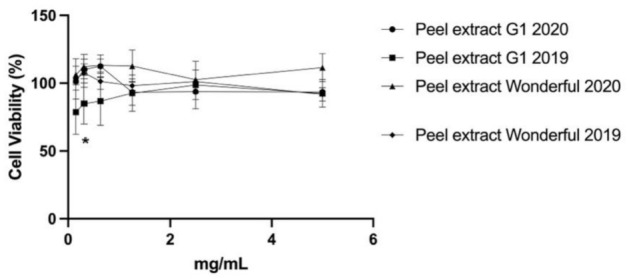
Cytotoxicity of the pomegranate peel extracts G1 and Wonderful 2019 and 2020 in HaCaT cells evaluated by MTT assay. For 24 h, cells were treated with an extract at different concentrations (0.15–5.00 mg/mL). The data are shown as a percentage of control cells and as the mean ± SEM of four separate experiments. (* *p* < 0.01 vs. untreated cells; one-way ANOVA with Dunnett post hoc test).

The cytocompatibility was evaluated in the pomegranate peel and male flower extracts 2019 and 2020 in keratinocytes HaCaT cells to find the nontoxic concentration of pomegranate extracts and further exploitation of these extracts as antimicrobial agents in cosmetics formulations. The use of keratinocytes was based on the concept that these cells play a significant role in creating skin structure and the sustenance of homeostasis, including the production of the skin barrier and the extracellular matrix (ECM). The cells were treated with extract concentrations ranging from 0.15 to 5.00 mg/mL for 24 h. The vitality of the cells was then determined by the MTT test. The results showed no significant difference in cell viability between the different pomegranate extracts (*p* > 0.05) varieties at any extract concentration. Figure 2 shows the cytotoxicity results obtained for the extracts selected with the most interesting antimicrobial properties, such as the G1 2020 and 2019 peel extracts and the Wonderful 2020 and 2019 peel extracts. Additionally, no change in the morphology was observed after treatment of keratinocytes with the extracts (data not shown).

Our results agreed with a previous study that reported null cytotoxicity of pomegranate extract against HaCaT cells [65]. Liu et al. [66] also demonstrated that the pomegranate extract resulted in no cytotoxicity in a concentration range from 6.25 to 100.00 µg/mL. These promising results obtained in the cytotoxicity tests confirm the suitability of the extracts as a natural booster ingredient for cosmetic formulations.

## 4. Conclusions

The main phenol components profile, antioxidant capacity, antibacterial activity, and cell viability of four pomegranate types cultivated in Marche were investigated. The UPLC-ESI-MS/MS study revealed that Mollar de Elche had a high concentration of punicalagin A and B, whereas G1 and Parfianka had high concentrations of gallic acid. Moreover, antibacterial activity testing revealed that all extracts were promising. Finally, this work shows the value of studying bioactive chemicals in agri-food waste products to generate innovative natural antibacterial and antioxidant components for cosmetic formulation. Further research will focus on the antibacterial activity of suitable pomegranate extract mixes and pomegranate extracts combined with conventional preservatives to minimise their concentration in cosmetic products.

## Figures and Tables

**Table 1:** Linearity, sensitivity, accuracy, and precision of the developed UPLC-ESI-MS/MS method for pomegranate peel and male flower by-products extracts.

Analytes	RT (min.)	Linearity ^a^		Sensibility		Accuracy ^d^	Precision ^e^
		Regression Curves	r^2^	LOD ^b^	LOQ ^c^		
Gallic acid	3.83	y = 109,036x	0.973	0.07	0.24	14.7	0.4–16.8
Punicalin	3.75	y = 115,291x	0.992	0.04	0.14	11.9	0.5–18.8
Punicalagin A	9.25	y = 139,898x	0.997	0.04	0.14	6.8	0.4–6.7
Punicalagin B	11.71	y = 149,562x	0.998	0.04	0.14	4.7	0.4–3.8
Ellagic Acid	21.84	y = 233,729x + 84,858	0.994	0.03	0.11	7.8	0.2–19.7
Cyanidin 3,5-diglucoside	6.2	y = 491,074x	0.998	0.08	0.24	3.0	3.6–14.4
Pelargonidin 3,5-diglucoside	8.4	y = 557,447x	0.999	0.05	0.14	1.6	0.5–13.2
Cyanidin 3-glucoside	10.3	y = 1,073,535.261	0.998	0.03	0.11	2.8	9.4–15.7
Pelargonidin 3-glucoside	12.0	y = 1,095,405x	0.993	0.01	0.03	1.7	0.2–18.6

^a^ explored in the concentration range of 1–50 mg/L for ellagic acid, gallic acid, punicalagin A and B, and punicalin; and in a concentration range of 1–20 mg/L in the case of cyanidin 3-glucoside, cyanidin 3,5-diglucoside, pelargonidin 3,5-diglucoside, and pelargonidin 3-glucoside, obtaining the corresponding calibration curve for further calculations. ^b^ Limit of detection. ^c^ Limit of quantification. ^d^ Accuracy was expressed as standard deviation %. ^e^ The precision was calculated for each compound, and it was expressed as relative standard deviation percentage. A range of SD% was reported in the table.

**Table 2:** Quantification of polyphenols by using UPLC-ESI-MS/MS. Each sample was isolated in triplicate and analysed separately. Three repetitions were used to determine the standard deviation.

Analyte	Wonderful Peel		Mollar de Elche Peel		G1 Peel		Wonderful Male Flowers		Mollar de Elche Male Flowers		Wonderful Peel		Mollar de Elche Peel		G1 Peel		Parfianka Peel	
	2019		2019		2019		2020		2020		2020		2020		2020		2020	
	Conc. ^1^	DS%	Conc. ^1^	DS%	Conc. ^1^	DS%	Conc. ^1^	DS%	Conc. ^1^	DS%	Conc. ^1^	DS%	Conc. ^1^	DS%	Conc. ^1^	DS%	Conc. ^1^	DS%
Gallic acid	9.7	12.6	33.6	2.2	17.2	1.7	925.2	5.2	789.5	1.4	28.5	1.9	47.8	13.1	53.3	16.8	58.5	0.4
Punicalin	7.7	0.5	34.1	18.8	<LOQ	<LOQ	5948.2	1.0	2143.8	3.3	638.7	1.2	946.4	3.7	670.1	8.1	67.6	3.1
Punicalagin A	478.9	6.5	2176.7	4.9	325.3	9.5	3562.2	0.4	430.4	1.32	2754.8	7.9	7206.4	4.6	3622.3	4.2	3767.3	6.7
Punicalagin B	947.8	1.9	3343.6	0.6	540.7	3.8	4757.8	0.5	667.5	3.3	3320.1	1.3	5812.9	1.3	2805.7	3.1	5367.8	0.4
Ellagic acid	48.9	12.8	231.2	0.4	19.7	6.6	42.4	2.4	87.1	3.5	418.9	0.2	289.7	2.7	337.3	1.5	123.2	3.0
Cyanidin 3,5-diglucoside	25.2	3.6	<LOQ	<LOQ	3.4	14.1	6.1	14.4	<LOQ	<LOQ	4.7	9.7	<LOQ	<LOQ	<LOQ	<LOQ	5.7	4.2
Cyanidin 3-glucoside	23.9	13.2	8.3	32.5	0.5	9.2	<LOQ	<LOQ	<LOQ	<LOQ	7.1	14.1	<LOQ	<LOQ	<LOQ	<LOQ	8.3	13.7
Pelargonidin 3,5-diglucoside	8.4	9.8	9.4	9.4	20.6	13.4	2.4	14.5	8.1	14.9	3.5	15.7	<LOQ	<LOQ	<LOQ	<LOQ	3.9	12.1
Pelargonidin 3-glucoside	13.1	18.6	7.2	18.6	1.8	0.2	1.1	14.4	<LOQ	<LOQ	7.0	10.1	<LOQ	<LOQ	<LOQ	<LOQ	8.1	13.2

^1^ The mean value is expressed as mg/Kg of DM (dry matter).

**Table 3:** Total phenol content (TPC) and antioxidant capacity (AC) of pomegranate extracts.

Samples	Folin–Ciocalteu	ABTS		FRAP	DPPH	
	(µmol GAE/g)	(µmol TEA/g)	IC_50(mg/mL)_	(µmol TEA/g)	(µmol TEA/g)	IC_50(mg/mL)_
Wonderful 2019 Peel	0.500 ± 0.004	0.076 ± 0.002	0.016 ± 0.001	2.170 ± 0.003	0.242 ± 0.056	0.065 ± 0.056
Mollar de Elche 2019 Peel	2.304 ± 0.006	3.290 ± 0.001	0.001 ± 0.001	3.299 ± 0.028	0.455 ± 0.007	0.035 ± 0.007
G1 2019 Peel	1.872 ± 0.002	2.121 ± 0.001	0.001 ± 0.001	3.730 ± 0.001	1.524 ± 0.012	0.011 ± 0.012
Wonderful 2020 male flowers	0.778 ± 0.003	6.808 ± 0.002	0.002 ± 0.001	0.615 ± 0.022	1.149 ± 0.014	0.014 ± 0.013
Mollar de Elche 2020 male flowers	0.746 ± 0.003	3.168 ± 0.002	0.001 ± 0.001	0.458 ± 0.013	0.444 ± 0.020	0.036 ± 0.023
Wonderful 2020 Peel	6.346 ± 0.001	29.301 ± 0.001	0.001 ± 0.002	7.015 ± 0.024	34.361 ± 0.001	0.001 ± 0.001
Mollar de Elche 2020 Peel	12.341 ± 0.002	18.862 ± 0.004	0.001 ± 0.001	12.435 ± 0.801	3.230 ± 0.003	0.003 ± 0.001
G1 2020 Peel	9.283 ± 0.015	21.754 ± 0.001	0.002 ± 0.001	12.407 ± 0.739	5.029 ± 0.010	0.002 ± 0.003
Parfianka 2020 Peel	6.098 ± 0.001	15.875 ± 0.001	0.001 ± 0.001	4.860 ± 0.237	4.393 ± 0.002	0.003 ± 0.001

**Table 4:** Evaluation of the antimicrobial activity by using *S. aureus*, *E. coli,* and *C. albicans.*

Samples	Diameter of	Evaluation ^a^
	Inhibition	
	(mm)	
*S. aureus*		
Wonderful 2019 Peel	8	++
Wonderful 2020 Peel	12	+++
Mollar de Elche 2019 Peel	<8	++
Mollar de Elche 2020 Peel	8	++
G1 2019 Peel	8	++
G1 2020 Peel	8	+
Parfianka 2020 Peel	8	+
Mollar de Elche Male flower	-	+
Wonderful Male flower	-	+
*E. coli*		
Wonderful 2019 Peel	10	
Wonderful 2020 Peel	10	+
Mollar de Elche 2019 Peel	10	
Mollar de Elche 2020 Peel	10	+
G1 2019 Peel	8	++
G1 2020 Peel	10	+
*C. albicans*		
Wonderful 2019 Peel	10	++
Wonderful 2020 Peel	10	++
Mollar de Elche 2019 Peel	10	++
Mollar de Elche 2020 Peel	12	++
G1 2019 Peel	12	+++
G1 2020 Peel	14	+++

^a^ The evaluation considers two parameters: the area of inhibition and the growth of inhibition, with + ranging from 0% to 40% with 1 mm, ++ ranging from 50% to 60% with 1 to 2 mm, and +++ ranging from 60% to 70% with >2 mm.

**Table 5:** Determination of IC_50_ against *S. aureus* and *P. aeruginosa* in G1 2020 peel extract.

Sample % ^a^	OD/mL	Rid. %
*S. aureus*		
0	0.4467	-
5	0.2733	23
10	0.2232	34
18	0.2932	20
20	0.2398	30
25	0.2318	32
30	0.1520	52
50	0.0052	97
*P. aeruginosa*		
0	0.7013	-
35	0.4600	34
40	0.3837	45
45	0.4630	34
50	0.4500	35

^a^ Tested sample dilution expressed in (%).

## Data Availability

Data is contained within the article and Supplementary Materials.

## Supplementary Materials

The following are available online at https://www.mdpi.com/article/10.3390/antiox11040768/s1, Table S1: Identification of polyphenols in pomegranate by-product extracts by UPLC-ESI-MS/MS analysis, Table S2: Identification of anthocyanins in pomegranate by-products extracts by UPLC-ESI-MS/MS analysis.
