# Supplementary material for: Chemical, Antioxidant, and Antimicrobial Properties of the Peel and Male Flower By-Products of Four Varieties of Punica granatum L. Cultivated in the Marche Region for Their Use in Cosmetic Products
Source: Antioxidants (Basel). 2022 Apr 12;11(4):768. doi: 10.3390/antiox11040768 (PMC9030693; doi:10.3390/antiox11040768)
Supplement: Supplementary file 1 [file antioxidants-11-00768-s001.zip › antioxidants-1675446-supplementary.pdf]

Supplementary Material

Chemical, antioxidant, and antimicrobial properties of the peel and male flowers by-products of four varieties of *Punica granatum* L. cultivated in the Marche region given their exploitation in cosmetic products

Summary of Tables

Table S1. Identification of polyphenols in pomegranate by-product extracts by UPLC-ESI-MS/MS analysis.

Table S2. Identification of anthocyanins in pomegranate by-products extracts by UPLC-ESI-MS/MS analysis.

Polyphenols identification

The first large group of hydrosysable tannins identified in our work contains three main subclasses of compound: gallotannins and ellagitannins belonging to the phenol groups that are esterified to the hydroxyl groups of glucose: gallic acid in gallotannins and hexahydroxydiphenic acid (HHDP) in ellagitannins [1] and gallagyl esters.

Compound **C1** and **C2** belonging to the classes of gallotannins showing a $[M-H]^-$ ions at m/z 331 and 483, respectively. **C1** reports a fragment ions in MS^2 at m/z 169 related to the loss of hexose fraction (162Da) and a fragment at m/z 125 which is typical for the fragmentation of gallic acid[M-H-162 Da-44 Da]. According to the literature **C1** was assigned to galloyl-hexoside, while **C2** was identified as digalloyl-hexoside according to its precursor ion at m/z 483 [2, 3].

Among hydrolysable tannins, ellagitannins compounds from **C3** to **C18** were identified. For this class of compound, characteristic fragments ions were identified as m/z 301 and attributed to the aglycone of ellagic acid that was obtained after a spontaneous-lactonization of the HHDP residue (**C16**) [3]. So, **C16** was identified as ellagic acid showing a typical signal at m/z 301and characteristic fragment ions at m/z 229 (corresponding to the loss of four H_2O molecules) and m/z 185 (corresponding to a loss of CO_2).

C3 at 2.3 min. produced a molecular ion at m/z 481 $[M-H]^-$ and was identified as HHDP-hex [3-5]. The ellagic acid hexoside moiety was also detected in the compounds C4, C11, C12, C13. **C4** showed an $[M-H]^-$ ion at m/z 633 and fragments at m/z 301 and 169 which are typical for the loss of ellagic acid and the galloyl moiety. **C11** showed an $[M-H]^-$ ion at m/z 935 and a typical fragments at m/z 633 and 301 and was identified as galloyl-bis-HHDPhexoside (casuaricitin) [3]. **C12** showed an [M-H]- ion at m/z 463 and typical fragments at m/z 301 indicated the loss of hexoside and was identified as ellagic acid hex[3, 4]. **C13** exhibited an $[M\_H]^-$ ion at m/z 951 producing fragments at m/z 933 and at m/z 301 (ellagic acid) in the MS2 experiment. This fragment (m/z 933), generating fragments at m/z 915 from the loss of water, is typical for castalagin/vescalagin or galloyl-gallagyl-hexoside (galloylpunicalin, pedunculagin III) as already reported in litterature [3, 5]. **C9** produced an $[M–H]^-$ ion at m/z 799 and fragments at m/z 479 (loss of ellagic acid), fragments at m/z 301(ellagic acid). Furthermore, the established fragments of ellagic acid were confirmed. This compound may be attributed to granatin A (HHDP-DHHDP-hexoside). **C6** (m/z1415) and C15 (m/z 433) produced fragments at m/z 933 and at m/z 301 (ellagic acid) in the MS2 experiment, respectively. C6 was identified as di(HHDP-galloylglucose)-pentose[6]. **C15** was assigned to an ellagic acid-pentoside that

in the MS2 experiment, the ion at m/z 301 was generated by the loss of 132 Da, reasonably assigned as the elimination of pentose. In addition, ellagitannins with a gluconic acid core were also found in pomegranates. Among these, **C5** was identified as galloyl-HHDP-gluconic acid. Its [M−H]− ion at m/z 649 showed fragments at m/z 497 and 301, resulting from the loss of gallic acid (releasing HHDP-gluconic acid) and ellagic acid, respectively. Galloyl-HHDP-gluconic acid (m/z 649) also formed part of **C10** exhibiting an [M−H]− ion at m/z 801. Further fragments at m/z 348 and 497 resulted from the loss of ellagic acid and gallic acid. This compound was identified as digalloyl-HHDP-gluconic acid (punigluconin). Compound **C7** exhibited an [M−H]− ion at m/z 783. The loss of ellagic acid in the MS2 experiment produced fragments at m/z 481 and the fragment atm/z 301 of ellagic acid. Based on this fragmentation pathway compound **C7** was identified as bis-HHDP-hexoside (pedunculagin I). **C8** was characterised as digalloyl-HHDP-hexoside (pedunculagin II). This assignment is based on its [M−H]− ion at m/z 785 and the release of typical ellagitannin and gallotannin fragments at m/z 301 (ellagic acid) and 169 (galloyl group). Each of the two different retention times corresponded to an isomeric structure, also differing in their fragmentation patterns. The [M-H]− ion of **C15** was obtained at m/z 433. In the MS2 experiment, the ion at m/z 301 was generated by the loss of 132 Da, reasonably assigned as the elimination of pentose. The occurrence of the ion at m/z 300 was attributed to a homolytic rupture of the glycosidic bond [5]. In addition to ellagic acid pentoside, further monoglycosylated ellagic acid derivatives were observed as an ellagic acid deoxyhexoside (m/z 447; **C14**) and an ellagic acid derivate (der) (m/z 441; **C17**) all of them showing the typical fragments of ellagic acid (m/z 301). An [M−H]− ion at m/z 469 was observed for the isomeric compound **C18**, producing fragment ions at m/z 425 indicating the loss of a carboxyl group and typical fragments of ellagic acid. Compound **C18** was therefore assigned to the valoneic acid bilactone [3]. **C19** eluted at 4.0 min with a precursor ion at m/z 781 and a fragment at m/z 601 (gallagyl residue) were identified as gallagyl-hexoside (punicalin). Identification of punicalagin was based on its MS spectrum which showed an exact $[M{-}H]^-$ ion at m/z 1083. Punicalagin α and β eluted in peaks **C20** (9.2 min) and **21** (11.7 min). MS spectra of both isomers showed a $[M{-}H]^-$ ion at m/z 1083, which fragmented at m/z 781 and m/z 601 (corresponding to the loss of ellagic acid and gallagic acid moiety, respectively). The 781 m/z peak **C 19** corresponded to the punicalin [M -H]- ion and a 302 m/z peak to the ellagic acid $[M\text{-}H]^-$ ion [7]. Gallic acid (**C22**) is a signal at m/z 169 and showed a typical fragment at m/z 125 corresponding to the loss of CO2. **C23** showed a signal at m/z 355 and fragment ions at m/z 193,175,217 and 236. This compound was identified as ferulic acid hex due to its fragmentation revealed the formation of an aglycone fragment at m/z 193 (ferulic acid) and fragments at m/z 175 from the loss of water. **C24** was identified as dihydrokaempferol-hex and produced an ion at m/z 449 and a fragments at m/z 287 (loss of hexose moiety), 259 and 269(loss of water). Among the class of gallotechin compounds **25** and **26** were identified as gallocatechin and catechin-gallocatechin, respectively. C25 showed a ion at m/z 306 producing a fragments at m/z 125,137,165 and 219, while C26 (m/z593) showed fragments at m/z 441,305,423 and 137 suggesting that the component of aglycone in these compounds was either catechin (290 Da) or epicatechin [2]. Compounds **C30** ansd **C31** showed an ion at m/z 447 reporting the same fragment ions at m/z 285 in the MS2 experiment. In case of **C30** were also detect the fragments ions at m/z 255 and 227 already detected fot the astralagin[2]. The **C31** were identified as luteolin-glucoside reporting a in its MS2 experiment, the fragment ions at m/z 285 which led to the identification of the aglycone as lutelolin [2]. **C29** (rt = 24.1 min) exhibited a $[M\text{-}H]^-$ ion at m/z 593. This compound was identified as kaempferol-7-orhahmano-glucoside due to in the MS2 experiment showed the fragment ion at m/z 285 and characteristic fragments at m/z 255, 227 belonging to kaempferol [2]. **C28** exhibited $[M\text{-}H]^-$ ions at m/z 609 with fragments ions at m/z 301 and 300 matched with the loss of rhamno-glucose (146 + 162 Da) and was identified as quercetin-3-O-rhahmano-glucoside (rutin) [2]. We also revealed the presence of **C27** (m/z 343, RT=1.54 min.) never found in pomegranate peel extracts, according to the literature. The identification of this compound will be further confirmed by HRMS analysis.

Table S1. Identification of polyphenols in pomegranate by-product extracts by UPLC-ESI-MS/MS analysis.

Peak number	R.T. (min.)	Precursor ion *m/z* [M-H]⁻	MS² ion *m/z*	Identification
**Hydrolysable tannins**				
Gallotannins				
C1	2.6	331	125,169	Galloyl-hex
C2	11.2	483	169,125,439	Digalloyl-hex
Ellagitannins				
C3	2.3	481	301,275,257	HHDP²-hex
C4	3.6	633	301, 275, 249, 169	Galloyl-HHDP-hex
C5	3.6	649	301, 497	Galloyl-HHDP-glucoside
C6	6.3	1415	633, 613, 783	Di(HHDP-galloylglucose)-pent
C7	6.8	783	301, 481, 275	Pedunculagina I
C8	11.7	785	301, 275, 249, 169	PedunculaginaII
C9	12.9	799	479, 301, 247	HHDP-DHHDP-hexoside Granatin A
C10	13.7	801	649, 348	Diggalloyl-HHDP-gluc (Punigluconin)
C11	13.8	935	633, 275, 708, 301	Galloyl-bis-HHDP-hex (Casuarinin)
C12	16.2	463	301, 300, 271, 255	Ellagic acid-hex
C13	19.3	951	301, 933, 273, 463	Galloyl-HHDP-DHHDP-hex (Granatin B)
C14	20.5	447	300, 301	Ellagic acid-deoxyhex
C15	20.7	433	300, 301	Ellagic acid-pent
C16	21.8	301	145,185,229	Ellagic acid
C17	22.4	441	397, 398, 301	Ellagic acid der
C18	24.2	469	425, 301, 426	Valoneic acid bilactone
**Gallagyl esters**				
C19	4.0	781	601, 721	Punicalin
C20	9.2	1083	601, 302, 781, 575	Punicalagin
C21	11.7	1083	601, 302, 781, 575	Punicalagin
**Hydroxybenzoic acids**				
C22	3.8	169	125	Gallic acid
**Hydroxycinnamic acids**				
C23	2.7	355	193, 175, 217, 236	Ferulic acid-hex
**Dihydroflavonol**				
C24	16.4	449	287, 259, 269	Dihydrokaempferol-hex
**Gallotechin**				
C25	6.8	306	125, 137, 165, 219	Gallocatechin
C26	7.0	593	441, 305, 423, 137	Catechin-gallocatechin
C27	1.54	343	181, 149, 113, 119, 89,59	Unknown
C28	3.2	609	301, 300, 271	Quercetin rutinoside
C29	24.1	593	285,255,227	Kaempferol rutinoside
C30	25.4	447	255,227,285	Astralagine
C31	26.3	447	285	Luteolin-glucoside

hex=hexoside; HHDP=hexahydroxydiphenoyl; pent=pentoside; der=derivate

The ESI source in positive ion mode was selected to detect anthocyanins extracted from pomegranate peel and male flowers extracts. MS and MS2 spectra of A1-A4 peaks showed

molecular ions characteristic of cyanidin (m/z 287), pelargonidin (m/z 271) confirmed by external standards. The anthocyanins revealed the typical mass spectrometric behavior in ESI(+)-experiments, i.e. they showed M $^{+}$experiments and the sequential loss of their saccharide moieties, releasing the aglycones in the MS+ ions.

Table S2. Identification of anthocyanins in pomegranate by-products extracts by UPLC-ESI-MS/MS analysis.

Peak number	R.T. (min.)	Precursor ion *m/z* [M]+	MS^2 ion *m/z*	Identification
A1	6.2	611	287, 449	Cyanidin 3,5-diglucoside
A3	8.4	595	433	Pelargonidin 3,5-diglucoside
A2	10.3	449	487	Cyanidin 3-glucoside
A4	12	433	271	Pelargonidin 3-glucoside

References

1. Bar-Ya'akovIrit; Tian Li; Amir Rachel; Holland Doron, Primary Metabolites, Anthocyanins, and Hydrolyzable Tannins in the Pomegranate Fruit. **2019,** 10, (620).
2. Abdulla Rahima; Mansur Sanawar; Lai Haizhong; Ubul Ablikim; Sun Guangying; Huang Guozheng; Aisa Haji Akber, Qualitative Analysis of Polyphenols in Macroporous Resin Pretreated Pomegranate Husk Extract by HPLC-QTOF-MS. *Phytochemical Analysis* **2017,** 28, (5), 465-473.
3. Fischer Ulrike A.; Carle Reinhold; Kammerer Dietmar R., Identification and quantification of phenolic compounds from pomegranate (Punica granatum L.) peel, mesocarp, aril and differently produced juices by HPLC-DAD–ESI/MSn. *Food Chemistry* **2011,** 127, (2), 807-821.
4. Hernández-Corroto Ester; Marina Mª Luisa; García Mª Concepción, Extraction and identification by high resolution mass spectrometry of bioactive substances in different extracts obtained from pomegranate peel. *Journal of Chromatography A* **2019,** 1594, 82-92.
5. Abid Mouna; Yaich Héla; Cheikhrouhou Salma; Khemakhem Ibtihel; Bouaziz Mohamed; Attia Hamadi; Ayadi M. A., Antioxidant properties and phenolic profile characterization by LC-MS/MS of selected Tunisian pomegranate peels. *J Food Sci Technol* **2017,** 54, (9), 2890-2901.
6. Mena Pedro; Calani Luca; Dall; Asta Chiara; Galaverna Gianni; García-Viguera Cristina; Bruni Renato; Crozier Alan; Del Rio Daniele, Rapid and Comprehensive Evaluation of (Poly)phenolic Compounds in Pomegranate (Punica granatum L.) Juice by UHPLC-MSn. *Molecules* **2012,** 17, (12).
7. Gosset-Erard Clarisse; Zhao Minjie; Lordel-Madeleine Sonia; Ennahar Saïd, Identification of punicalagin as the bioactive compound behind the antimicrobial activity of pomegranate (Punica granatum L.) peels. *Food Chemistry* **2021,** 352, 129396.
